# Enhanced Bone-Defect Regeneration Through nHA/Chitosan Nanocomposite-Facilitated Delivery of HUCB-MSCs-Derived Exosomes

**DOI:** 10.3390/polym18131562

**Published:** 2026-06-23

**Authors:** Lingzhi Ding, Jiachen Liu, Jia Gao, Yongqian Fu, Wenhui Chu, Shunwu Fan

**Affiliations:** 1 Department of Orthopaedics, Sir Run Run Shaw Hospital, School of Medicine, Zhejiang University, Hangzhou 310000, China; 2 Taizhou Key Laboratory of Biomass Functional Materials Development and Application, School of Life Science, Taizhou University, Taizhou 318000, China; 3 College of Biotechnology and Pharmaceutical Engineering, Nanjing Tech University, Nanjing 211816, China

**Keywords:** chitosan, exosomes, bone tissue engineering

## Abstract

Critical-sized bone defects lack spontaneous healing capacity. While mesenchymal stem cell-derived exosomes (sEVs) are promising osteoinductive agents, their rapid in vivo clearance limits their free-form efficacy. Here, we fabricated a nano-hydroxyapatite/chitosan (nHA/CTS) composite scaffold as a protective, sustained-delivery platform for human umbilical cord blood-derived mesenchymal stem cell exosomes (HUCB-MSCs-exos) to accelerate bone repair. The 3D porous CTS/10% nHA scaffold exhibited excellent cytocompatibility and a degradation rate commensurate with new bone ingrowth. Critically, it enabled a biphasic exosome release profile—an initial burst followed by a 14-day sustained release (89.73% cumulative release). In vitro, HUCB-MSCs-exos significantly promoted the proliferation, migration, and osteogenic differentiation of bone marrow-derived MSCs, as demonstrated by enhanced alkaline phosphatase activity and matrix mineralization. In a rabbit condylar defect model (5 mm diameter), the CTS/10% nHA-exo scaffold achieved a 57.44 ± 8.42% healing rate at two months, nearly two-fold greater than the scaffold-only group (29.33 ± 6.94%). Histological and immunohistochemical analyses at two months confirmed the formation of mature, well-vascularized trabecular bone, accompanied by robust expression of late-stage osteogenic markers (OCN and OPN). These findings demonstrate that the CTS/10% nHA scaffold synergistically integrates osteoconductive structural guidance with exosome-mediated osteoinductive paracrine signaling, providing a compelling and translatable strategy for critical-sized bone-defect management.

## 1. Introduction

Bone defects are a prevalent clinical condition characterized by compromised structural integrity and significant bone loss resulting from severe trauma, tumor resection, or infections [1]. As the global incidence of bone-related injuries and disorders continues to rise annually [2], the clinical demand for effective treatments remains substantial. Although bone tissue possesses an intrinsic capacity for regeneration, critical-sized defects exceeding a specific threshold cannot heal spontaneously [3]. Currently, the gold standard treatments for repairing such defects involve autologous or allogeneic bone grafting and the use of bone substitutes [4]. However, these approaches are hampered by inherent limitations, including postoperative infections, immune rejection, inadequate mechanical properties, and donor-site morbidity [5]. Consequently, there is a compelling need to explore safer and more efficacious strategies that not only promote osteogenesis in deficient areas but also facilitate the reconstruction of the microcirculatory system between the graft and the host—a widely recognized prerequisite for successful bone remodeling.

Mesenchymal stem cells (MSCs) are multipotent stromal cells characterized by their self-renewal and multilineage differentiation capabilities. Isolated from diverse sources such as human term placenta [6], bone marrow [7], adipose tissue [8], and urine [9]. MSCs offer distinct advantages, including potent immunomodulatory properties, targeted tissue repair, high biocompatibility, and low immunogenicity [10]. While their broad applicability in regenerative medicine is well documented [11], human umbilical cord blood-derived MSCs (HUCB-MSCs) are particularly advantageous, as they maintain a low immunogenic phenotype under specific biological conditions, thereby facilitating the development of “off-the-shelf” products for clinical transplantation [12]. Nevertheless, direct transplantation of live stem cells is constrained by severe translational bottlenecks, such as poor survival rates in ischemic microenvironments, potential tumorigenicity, and logistical challenges associated with mass storage and transportation [13]. As a result, “cell-free therapy” has emerged as a safer and more stable alternative.

Emerging research indicates that MSCs exert their therapeutic efficacy primarily through paracrine mechanisms, which are essential for maintaining cellular homeostasis [5]. Exosomes, a key component of this paracrine secretion, are cell-derived extracellular vesicles (30–150 nm in diameter) encompassing a cargo of lipids, proteins, and nucleic acids [14]. By transferring these bioactive molecules to recipient cells, exosomes facilitate critical intercellular communication [15]. Notably, stem cell-derived exosomes have been shown to regulate angiogenesis, apoptosis, and immune responses, while playing a pivotal role in driving osteoclast and osteoblast differentiation via paracrine signaling, thus making them highly promising agents for bone repair [16]. Despite their immense therapeutic potential, the clinical utility of naked exosomes administered via direct local injection is severely restricted by their rapid clearance through the reticuloendothelial system (RES) and bodily fluids, resulting in a drastically shortened in vivo half-life. Therefore, engineering a structural carrier capable of shielding exosomes and enabling their sustained, localized release is urgently required to advance exosome-based bone tissue engineering [5,17].

Ideal bone scaffolding materials must exhibit excellent biocompatibility, controlled degradability, and appropriate mechanical strength to support cell adhesion, proliferation, and de novo bone formation [18]. Nano-hydroxyapatite (nHA), owing to its low crystal repeat period, reduced crystallinity, and nanoscale dimensions, demonstrates superior in vivo degradation and absorption compared to conventional hydroxyapatite [19]. Furthermore, its optimized surface properties facilitate the adsorption of specific proteins, enhancing bioactivity and augmenting osteoblast function [20]. However, the clinical application of pure nHA is significantly hindered by its inherent brittleness, poor moldability, and propensity to elicit inflammatory responses due to the presence of irregular or high-density particles [21]. To overcome these limitations, composite scaffolds combining nHA with natural polymers, such as chitosan (CTS), have been extensively explored [22]. CTS is a natural cationic carbohydrate polymer renowned for its biodegradability, non-immunogenicity, and excellent adsorption capacity [23]. Its surface hydrophilic functional groups promote robust cell adhesion, making it an exceptional carrier for sustained drug delivery. Modifying nHA with CTS not only generates uniformly fillable microspheres but also mitigates inflammation and stimulates osteogenesis and matrix mineralization [24,25]. More importantly, the porous network and cationic nature of the chitosan/nano-hydroxyapatite (CTS/nHA) composite make it an ideal biomaterial vehicle for electrostatically encapsulating negatively charged exosomes, shielding them from premature degradation while providing the essential three-dimensional (3D) osteoconductive template for bone ingrowth [26].

**Scheme 1:**
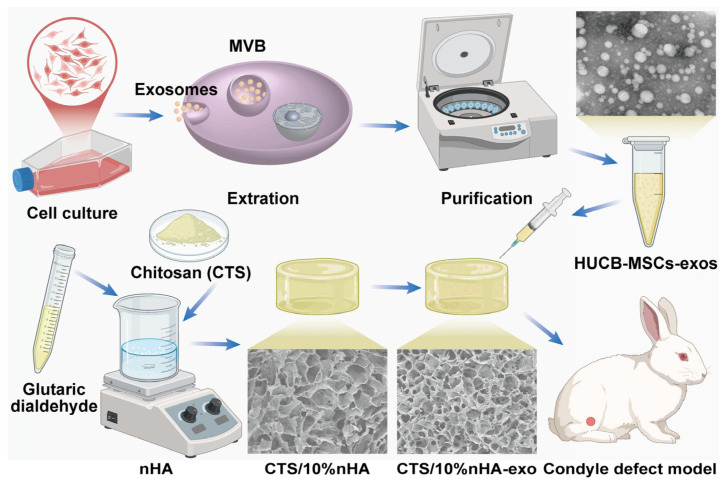
Schematic illustration of the study.

While various scaffold and hydrogel systems have been widely utilized for the delivery of MSC-derived exosomes in bone regeneration [27,28], the clinical translation of these platforms often suffers from mismatched degradation rates and suboptimal release kinetics. Previous exosome-loaded CTS/nHA scaffolds have demonstrated osteogenic potential [29]; however, achieving a spatiotemporal coordination between scaffold resorption, prolonged bioactive cargo release, and the native bone healing cascade remains a formidable challenge. To address these specific bottlenecks, we engineered a CTS/nHA scaffold functionalized with HUCB-MSC-derived exosomes (CTS/nHA-exo). We hypothesize that the CTS/nHA composite will act as a biomimetic osteoconductive matrix capable of delivering a sustained release of exosomes, thereby orchestrating a microenvironment highly conducive to concurrent osteogenic differentiation and vascularization. To systematically evaluate this paradigm, we first investigated the in vitro effects of these exosomes on the proliferation and osteogenic differentiation of bone marrow mesenchymal stem cells (BMSCs). Subsequently, the engineered CTS/10% nHA-exo nanoscaffolds were implanted into a rabbit condylar defect model (Scheme 1). The novelty of this platform is anchored in the synergistic integration of three key elements. Primarily, we strategically selected HUCB-MSC exosomes over adult tissue-derived exosomes, capitalizing on their inherently low immunogenicity and superior osteo-angiogenic properties for “off-the-shelf” clinical applicability. Furthermore, precise calibration of the composite to exactly 10% nHA ensures that the scaffold’s degradation kinetics closely match the pace of native bone regeneration, maintaining essential structural integrity. Most importantly, this compositional tuning unlocks a unique biphasic release profile. This enables a rapid initial exosome burst for immediate progenitor cell recruitment, smoothly transitioning into a 14-day sustained delivery that persistently directs both osteogenic and vascular differentiation. By synergistically combining the structural advantages of inorganic-organic nanocomposites with the potent paracrine signaling of stem cell-derived vesicles, this study aims to establish a robust, off-the-shelf “cell-free” platform for targeted bone regeneration and subsequent clinical translation.

## 2. Materials and Methods

### 2.1. In Vitro Multipotent Differentiation Assay of HUCB-MSCs and BMSCs

#### 2.1.1. Osteogenic and Adipogenic Differentiation

The HUCB-MSCs were obtained from Wuhan Ponsure Life Science and Technology Co., Ltd. (Wuhan, China). Rabbit BMSCs were obtained by Guangzhou Xinyuan Biological Co., Ltd. (Guangzhou, China). The HUCB-MSCs and BMSCs were seeded into 6-well plates (1 × 10^5^ cells/well) and incubated with 2 mL of Cellartis^®^ MSC Xeno-Free Culture Medium (TAKARA, Shiga, Japan). After HUCB-MSCs and BMSCs reached approximately 70% confluence, the cell medium was replaced with osteogenic induction medium: high glucose-DMEM (Gibco, Waltham, MA, USA) containing 10% FBS, 10 nM dexamethasone (Beyotime, Shanghai, China), 10 mM β-glycerol phosphate (Sigma, Louis, MO, USA), 1.8 mM tripotassium orthophosphate (Macklin, Shanghai, China), and 50 μg/mL L-ascorbic acid (Beyotime, Shanghai, China). The medium was refreshed every 3 days. After 21 days, the cells were fixed and assayed using the Alizarin Red Staining (ARS) Kit for Osteogenesis (Beyotime, Shanghai, China). For adipogenic differentiation, the HUCB-MSCs and BMSCs were cultured in adipogenic differentiation medium: high glucose-DMEM containing 1 μM dexamethasone, 0.1 mM isobutylmethylxanthine (Sigma, Louis, MO, USA), 200 μM indomethacin (Sigma, Louis, MO, USA), and 10 mg/mL insulin (MCE, Monmouth Junction, NJ, USA). After 21 days, adipogenesis was assessed by Oil Red O staining (Beyotime, Shanghai, China). The stained cells were then observed under an inverted microscope (TSI-1000, TUSEN, Shanghai, China).

#### 2.1.2. Immunofluorescence Staining

HUCB-MSCs and BMSCs on coverslips were fixed in 4% paraformaldehyde (PFA) at 4 °C for 30 min, then soaked in blocking solution (0.5% Triton X-100 with phosphate-buffered saline (PBS) in a 1:1 ratio, then added 10% serum). Then, the cells were incubated with primary antibody against CD44 (rabbit anti-CD44 antibody, diluted in PBS at 1:100, Proteintech, Rosemont, IL, USA) overnight at 4 °C. After washing with PBS, the cells were incubated with secondary antibody (Goat anti-Rabbit IgG (H + L) Cross-Adsorbed Secondary antibody, Alexa Fluor 488, 1:500) at room temperature for 2 h in the dark, then with DAPI solution (1:1000, Solarbio, Beijing, China) in the dark for an additional 5 min and washed. Finally, a drop of Fluoromount-G (0100-01, SouthernBiotech, Birmingham, AL, USA) was placed on the slide, and the slide was covered with cells. Images were collected via an inverted fluorescence microscope (ECLIPSE Ti-U, Nikon, Tokyo, Japan).

#### 2.1.3. Flow Cytometric Analysis

The surface antigens of HUCB-MSCs (iCell Bioscience Inc., Shanghai, China) were analyzed by flow cytometry. Passage 3 and passage 6 (P3 and P6) cells were adjusted to 1.8 × 10^6^ cells/mL using FACS buffer (PBS with 2% FBS) and filtered through a 40 µm disposable cell strainer to remove cell clumps. Subsequently, cells were centrifuged at 1000 r/min for 5 min, washed with PBS twice, and aliquoted into four 1.5 mL Eppendorf tubes. One tube served as a control without antibodies, while 2 µL of antibodies (CD73-PE, CD90-PE-Cy7, CD105-APC) were, respectively, added to other Eppendorf tubes. After mixing, the cells were incubated at 4 °C in the dark for 30 min. The cells were washed twice with 1 mL PBS, each time followed by centrifugation at 1000 r/min for 5 min, and the supernatant was discarded. Finally, the cell pellet was resuspended in 400 µL PBS, transferred to flow cytometry tubes, and analyzed with a FACSAria II flow cytometer (Becton Dickinson, Franklin Lakes, NJ, USA).

### 2.2. Isolation, Characterization, and Cellular Uptake of HUCB-MSCs-Exos

Exosomes were isolated from HUCB-MSCs using the Cell Culture Supernatant Exosome Extraction Kit (Solarbio, Beijing, China). HUCB-MSCs were cultured in Cellartis^®^ MSC Xeno-Free Culture Medium. When HUCB-MSCs reached 70~80% confluence, the cultured media containing HUCB-MSCs was collected and centrifuged at 3000× *g* for 15 min to remove cellular debris. Following, 5 mL of exosome concentration solution was added to 20 mL of culture medium, and the mixture was kept at 4 °C for 12 h. A mixture of exosome concentration solution and cultured media was centrifuged at 10,000× *g* for 1 h, removing the supernatant and resuspending in PBS. The supernatant was transferred to a new tube and centrifuged at 12,000× *g* for 2 min, and then the exosomes were purified using purification columns. Finally, exosomes dissolved in PBS were stored at −80 °C until use.

Transmission electron microscopy (TEM, HT7700, Hitachi, Tokyo, Japan) was used to observe the morphology of exosomes. The particle size distribution of the exosomes and lyophilized exosomes was measured by Particle Size & Zeta potential Analyzer (Zetasizer Nano ZS90, Malvern, UK).

The exosomes were labeled with PE-CD9 (PE-anti-human-CD9, BioLegend, San Diego, CA, USA). Briefly, 2 μL of PE-CD9 was added to the exosome suspension, and the mixture was incubated for 30 min at 4 °C. Then, BMSCs were seeded into confocal dishes, the fluorescent dye suspension was added, and the cells were placed in a cell culture incubator for further cultivation, and then labeled with Hoechst 33342 (Beyotime, Shanghai, China). The confocal dishes were detected by laser scanning confocal microscopy (LSCM, Olympus, Tokyo, Japan) at 1 h, 3 h, and 6 h, respectively.

### 2.3. Synthesis of CTS/nHA and CTS/10% nHA-Exo

CTS/nHA was prepared by physical blending. Briefly, nHA of different weights was dissolved in 36 mL of 2% acetic acid solution and sonicated for 15 min on ice to obtain a grayish-white suspension. A total of 0.8 g of CTS was added to the nHA solution to make the CTS/nHA weight ratios 0, 5, 10, 15, 20, 25, 30, 50, and 100, respectively; then the solution was mechanically stirred for 2 h. Then, 0.25% glutaraldehyde was added to CTS solutions and stirred for 30 min to crosslink the CTS matrix. The mixtures were freeze-dried to form scaffolds. Afterward, these scaffolds were immersed in NaOH solution for 12 h and then washed with deionized water until the pH became neutral. Finally, all the neutralized scaffolds were lyophilized again. We ultimately selected CTS/10% nHA to load exosomes. According to previous studies [30,31], to immobilize the exosomes, the scaffolds were immersed in a 1 mg/mL exosome solution for 12 h at 4 °C to adsorb the exosomes.

### 2.4. Characterization of Scaffolds

The surface morphology and pore dimensions of the scaffolds were analyzed using scanning electron microscopy (SEM, S-4800, Hitachi, Tokyo, Japan). The elemental distribution of the scaffold was obtained by energy-dispersive spectroscopy (EDS, S-4800, Hitachi, Japan). The functional groups of CTS/10% nHA and CTS/10% nHA-exo were analyzed by an IS10 spectrometer (Thermo Fisher Scientific, Waltham, MA, USA) using KBr pellets, scanned from 4000 to 400 cm^−1^.

### 2.5. In Vitro Degradation and Swelling Ratio of CTS and CTS/10% nHA

The synthesized scaffolds were tested using lysozyme for biodegradability. Specifically, CTS and CTS/10% nHA were soaked in lysozyme in PBS for 2 weeks at 37 °C. The initial scaffold samples were weighed and recorded as initial mass (W_i_). At predetermined time points, scaffold samples were rinsed with ddH_2_O and then dried for 12 h at 40 °C to remove the water. After drying, scaffolds were weighed again and recorded as the final mass (W_t_). Each sample was replicated three times; the degradation was calculated according to the formula below:

Degradation (%) = [(W_i_ − W_t_)/W_t_] × 100

The swelling studies were performed in PBS, SPB, and ddH_2_O. The dry weight of the scaffold was noted (W_0_). CTS/10% nHA scaffold was incubated in PBS, simulated body fluid (SBF), and ddH_2_O for 24 h at room temperature until swelling equilibrium state. Afterward, the scaffolds were taken out of the solution, and the adsorbed water on the surface was drained by filter paper. Then, the wet weight was recorded (W_w_). The ratio of swelling was determined using the formula:

Swelling ratio (%) = [(W_w_ − W_0_)/W_0_] × 100

### 2.6. Mechanical Properties of CTS and CTS/10% nHA

CTS and CTS/10% nHA scaffolds were prepared into cylinders approximately 7.16 mm in diameter and 9 mm in height, with three parallel samples set for each group. The scaffolds were sequentially placed in the center of the sample stage of a universal mechanical testing machine (UTM2502, Senstest, Shenzhen, China). A uniaxial compression test was performed with the strain rate set at 5 mm/min and a preload of 0.2 N. Pressure was gradually applied to measure the elastic modulus, compressive strength, and compressive strain of the CTS and CTS/10% nHA scaffolds.

### 2.7. Cytocompatibility Assessment and Release Profile of CTS/10% nHA

BMSCs (Osteoblast cell line) were cultured in Cellartis^®^ MSC Xeno-Free Culture Medium and incubated at 37 °C, humidified atmosphere with 5% CO_2_. The scaffolds were washed with sterile PBS containing 1% penicillin streptomycin several times, then placed into a 12-well plate. An adequate amount of culture medium was added to cover the scaffold surface. After 24 h at 37 °C, the leaching solution was collected. Cells were inoculated into a 96-well plate; after cell adhesion, the culture medium was removed, and the above leaching solution was added. Use the same conditions to culture the control group with empty medium for 24 h or 48 h, filter-sterilize under the same conditions, culture simultaneously, and perform the CCK-8 assay.

To determine the release profile of HUCB-MSCs-exos, the CTS/10% nHA-exo scaffolds were incubated in PBS at 37 °C, and the supernatant was collected at predetermined time intervals. The protein content of the supernatant was tested by a BCA protein assay kit (Beyotime, Shanghai, China).

### 2.8. Cell Proliferation and Migration Scratch

Four different concentrations of HUCB-MSCs-exos (0, 10, 20, and 30 µg/mL) were applied to evaluate the impact on BMSCs’ proliferation. In brief, BMSCs (5000 cells/well) were seeded into 96-well plates and cocultured with different concentrations of exosomes (10, 20, and 30 µg/mL) for 24 h and 48 h. The CCK-8 kit (Beyotime, Shanghai, China) was used to evaluate cell proliferation, with absorbance measured at 450 nm.

To assess the cell migration capability, BMSCs were seeded into the cell scratch inserts at a density of 1 × 10^6^ cells/well with 70 µL added to each well. Once the confluency reached 100%, the inserts were taken out, and solutions of different concentrations of HUCB-MSCs-exos were added. The cell migration process was recorded at 0 h, 12 h, and 24 h after scratching, and stained with crystal violet for 10 min after 24-h incubation. Migration was quantified by measuring the residual fractional wound area using the ImageJ 1.53t software.

### 2.9. In Vitro Osteogenesis

MC 3T3-E1 cells (Taiyuan Rosetta Stone Biotech Co., Ltd., Taiyuan, China) were chosen to evaluate the osteogenesis differentiation ability of HUCB-MSCs-exos. MC 3T3-E1 cells were seeded into 96-well plates at a density of 5 × 10^3^ cells/well. After overnight attachment, the cells were rinsed with PBS and incubated with the native control (NC) medium (DMEM containing 10% FBS and 100 U/mL penicillin streptomycin), the positive control (PC) medium (osteogenic induction medium: DMEM containing 10% FBS, 100 U/mL penicillin streptomycin, 10 nM dexamethasone, 10 mM β-glycerol phosphate, 1.8 mM tripotassium orthophosphate and 50 μg/mL L-ascorbic acid), along with exosomes modified osteogenic induction medium (with exosomes concentrations of 10, 20 and 30 μg/mL, respectively), which were refreshed every 2 days. On day 14, the alkaline phosphatase (ALP) activity assay was performed using the BCIP/NBT alkaline phosphatase color development kit (Beyotime, Shanghai, China) and an ALP assay kit. After culturing for 21 days, the mineralized matrix was assessed using the Stem Cell Osteogenic Differentiation Potential Colorimetric Quantification Assay Kit (Yuduo, Shanghai, China) and the Alizarin Red Staining Kit for Osteogenesis (Beyotime, Shanghai, China). The stained cells were observed under a Stereomicroscope (M205FCA, Singapore).

### 2.10. Surgical Procedure of the Rabbit Condyle Defect Model

Approval for all experimental procedures was obtained from the animal ethics committee of Taizhou University (TZXY-2024-20241106). All the female New Zealand white rabbits (2-month-old and weighing 3.0 ± 0.3 kg) were fed in animal housing conditions and provided adequate material, water, and chow. As illustrated in Figure S1, to establish the rabbit condylar defect model in accordance with a previously described protocol [31], anesthesia was initially induced via an auricular intravenous injection of propofol (15 mg/kg) and subsequently maintained with isoflurane; following anesthetic induction, the surgical sites of all animals were shaved and systematically disinfected using 75% (*v*/*v*) ethanol and iodine. Then, a longitudinal incision was made on the lateral sides of the rabbit knee bones. The subcutaneous tissue and muscles were directly separated to expose the condyle bone. The condyle bone defect was created in the center of the condylar bone with a length of 10 mm and a diameter of 5 mm using a power drill. The rabbits were randomly divided into 5 groups: (A) blank control group, (B) CTS scaffold group, (C) CTS/10% nHA scaffold group, (D) CTS/10% nHA-exo scaffold group, and (E) bone cement group. Each group had the corresponding scaffold material implanted (n = 6). The surgical incisions were closed, and penicillin streptomycin was injected subcutaneously to prevent infection. Two months after surgery, rabbits from each group were sacrificed, and the collected tissue samples were immersed in a decalcifying solution for further analysis.

### 2.11. CT and Histological Evaluation

The specimens were scanned using a CT system (Sensation 64, Siemens Healthineers, Forchheim, Germany) to observe the new bone formation at the defect sites. The scans were performed with the following settings: 120 kV, 160 mA, 0.6 mm of slice thickness, and 0.7 s/Rot of rotational speed. Then the images were reconstructed using the RSVS 8.1.V.Lite software and evaluated by the Image J 1.54p software.

For histological observation, the bones were decalcified for 10 days and then dehydrated with a graded series of ethanols. Subsequently, the samples were cleared in xylene and embedded in paraffin. Tissue slices with a thickness of 6 μm were cut from the cross-section of the central area of each defect and stained with H&E and Masson’s trichrome for microscopic evaluation [32].

To further evaluate the bone formation at the bone-defect sites, immunohistochemical staining was performed for osteogenesis-related expression factors: osteopontin (OPN) and osteocalcin (OCN). Specifically, 6 μm tissue sections were dewaxed, rehydrated, and subjected to heat-mediated antigen retrieval [32]. The sections were then treated with 3% hydrogen peroxide to block endogenous peroxidase. After washing with PBS, the sections were blocked with 10% normal goat serum for 30 min at room temperature. Next, the sections were incubated with primary antibodies (Table 1) overnight at 4 °C. Following PBS washing, the sections were incubated with HRP-conjugated secondary antibodies at 37 °C for 2 h and counterstained with hematoxylin before being observed under a microscope. All the images were recorded with an inverted microscope with a digital CaseViewer.

### 2.12. Statistical Analysis

All data obtained from the studies were evaluated statistically using the GraphPad Prism 8.0.2 software and presented with mean ± standard deviation (SD) unless otherwise indicated. Student’s *t*-test or analysis of variance (ANOVA) was applied for comparison between two groups or among multiple groups, respectively, to determine the presence of any significant difference between groups. (* *p* < 0.05, ** *p* < 0.01, *** *p* < 0.001, **** *p* < 0.0001).

## 3. Results and Discussion

### 3.1. Characterization of HUCB-MSCs and BMSCs

The morphology of cultured HUCB-MSCs and BMSCs, observed under a stereo microscope, is illustrated in Figure S2. Trilineage differentiation assays and flow cytometry analyses were subsequently performed to comprehensively characterize both cell populations. As shown in Figure S2, both HUCB-MSCs and BMSCs successfully differentiated into adipocytes and osteoblasts under their respective induction conditions, thereby demonstrating robust multipotent differentiation capacity.

Immunofluorescence staining for CD44, a well-established surface marker of MSCs [33,34], further confirmed the mesenchymal identity and origin accuracy of both HUCB-MSCs and BMSCs (Figure S3). Moreover, quantitative flow cytometry revealed that CD73-PE, CD90-PE-Cy7, and CD105-APC were highly expressed in passage 3 (P3) HUCB-MSCs, with uniformly positive staining across all three markers (Figure S4). Taken together, these findings collectively confirm that the differentiation potential and surface marker phenotype of HUCB-MSCs and BMSCs are fully consistent with established characterization criteria reported in the literature [35,36]. This rigorous validation of the parental cells constitutes a critical prerequisite for downstream studies, as the regenerative efficacy and osteoinductive cargo of MSC-derived exosomes are profoundly influenced by the physiological state and purity of their cellular source.

### 3.2. Characterization of HUCB-MSCs-Exos

**Figure 1:**
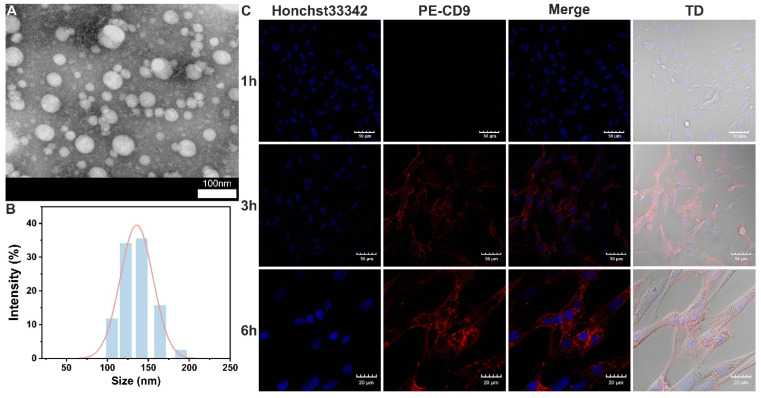
(**A**) TEM images of HUCB-MSCs-exos. Scale bar, 100 nm. (**B**) Particle size analysis of HUCB-MSCs-exos. (**C**) Uptake of exosomes by BMSCs. Scale bar: 50 μm and 20 μm.

In accordance with the Minimal Information for Studies of Extracellular Vesicles (MISEV) guidelines [37], the isolated HUCB-MSCs-exos were comprehensively characterized by morphology, size distribution, and specific protein markers. TEM revealed that the HUCB-MSCs-exos exhibited classic cup-shaped, circular, or elliptical morphology with intact lipid bilayer membranes (Figure 1A). Nanoparticle tracking analysis (NTA) demonstrated a unimodal size distribution with a peak diameter of approximately 136 nm (Figure 1B), a size range considered highly favorable for cellular internalization via endocytic pathways and thus conducive to the efficient intracellular delivery of exosomal cargo [38].

The uptake dynamics of these exosomes by recipient BMSCs were monitored using LSCM (Figure 1C). The results showed that exosomes labeled with PE-anti-human-CD9 (red fluorescence) were progressively internalized by BMSCs over a time course of 1, 3, and 6 h. Notably, after 3 h of co-incubation, substantial accumulation of exosomes was observed predominantly in the perinuclear region (delineated by DAPI-stained nuclei, blue fluorescence). This characteristic perinuclear localization is of considerable functional significance, as it suggests that the exosomes successfully deliver their regulatory cargos—including osteogenic miRNAs and growth factors—to the vicinity of the host cell nucleus, thereby positioning them to modulate gene transcription and orchestrate the osteogenic differentiation of BMSCs [39]. Furthermore, this fluorescence colocalization assay confirmed that the isolated nanovesicles abundantly express the classical exosomal transmembrane tetraspanin marker CD9 [7,16], verifying the successful isolation of exosomes capable of mediating functional intracellular communication.

### 3.3. Fabrication and Physicochemical Characterization of CTS/10% nHA Composite Scaffolds

**Figure 2:**
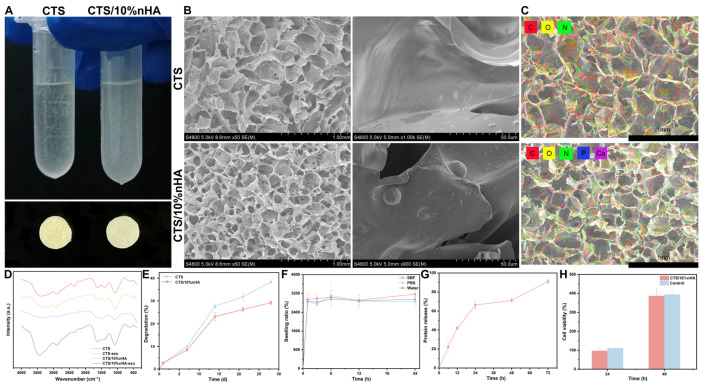
Characterization of scaffolds. (**A**) Images of the CTS and CTS/10% nHA before and after freeze-drying. (**B**) SEM of CTS and CTS/10% nHA. Scale bar, 1 mm and 50 μm. (**C**) EDS assay of CTS and CTS/10% nHA. Scale bar, 1 mm. (**D**) FTIR spectra of CTS, CTS-exo, CTS/10% nHA, and CTS/10% nHA-exo. (**E**) The degradation kinetics of CTS and CTS/10%nHA immersed in lysozyme. (**F**) The swelling dynamics of CTS/10% nHA in PBS, SBF, and Water. (**G**) In vitro exosomes release kinetics in PBS from CTS/10% nHA-exo. (**H**) Cell proliferation assay of CTS/10% nHA at 24 h and 48 h.

Among the various CTS/nHA formulations evaluated at different ratios, CTS/10% nHA exhibited the optimal overall performance in terms of mechanical integrity, porosity, and structural homogeneity (Figure S6); this ratio was therefore selected for all subsequent investigations. The macroscopic morphology of CTS and CTS/10% nHA scaffolds before and after freeze-drying is presented in Figure 2A, confirming successful fabrication with a well-maintained three-dimensional architecture.

#### 3.3.1. Structural and Compositional Analysis

SEM images of CTS, CTS-exo, CTS/10% nHA, and CTS/10% nHA-exo scaffolds are presented in Figure 2B and Figure S7. All scaffolds displayed well-interconnected, three-dimensional porous architectures that are essential for bone tissue engineering, as they facilitate not only the efficient removal of metabolic waste and cellular debris but also the transport of oxygen, nutrients, and osteogenic signaling molecules throughout the construct [40]. Importantly, the incorporation of small amounts of nano-hydroxyapatite did not alter the porous microstructure of the scaffold, and SEM analysis further confirmed that exosome loading preserved the original scaffold morphology. The morphology of exosomes observed on the scaffold surface is consistent with that reported in prior studies [7,41,42]. EDS mapping further corroborated the successful integration of nHA into the scaffold; compared with pure CTS scaffolds, the CTS/10% nHA scaffolds exhibited distinct calcium (Ca) and phosphorus (P) signals attributable to the incorporated nano-hydroxyapatite (Figure 2C and Figure S8), confirming uniform adhesion and distribution of nHA particles across the scaffold surface.

Fourier Transform Infrared (FTIR) spectroscopy was employed to evaluate the chemical structure of CTS, CTS/10% nHA, and their exosome-loaded counterparts (Figure 2D). In the pristine CTS spectrum, the broad absorption band at 3410 cm^−1^ corresponds to the stretching vibration of hydroxyl (–OH) groups overlapping with the –NH_2_ stretching vibration, while the bands in the range of 1610–1538 cm^−1^ are attributed to the C=O stretching vibration (Amide I) and the N–H in-plane bending vibration (Amide II), respectively. Additional characteristic peaks were identified at 2890 cm^−1^ (–CH stretching vibration), 1383 cm^−1^ (–CH_3_ and –CH_2_ bending vibrations), and 1079 cm^−1^ (C–O–C stretching vibration). For the CTS/10% nHA composite, bands at 3428, 1612, and 626 cm^−1^ were assigned to the stretching and bending vibrations of hydroxyl groups in hydroxyapatite, and the bands at 600 and 536 cm^−1^ correspond to the symmetric stretching vibration of phosphate (PO_4_^3-^) groups, consistent with the characteristic fingerprint of crystalline hydroxyapatite reported in the literature [35,43]. These spectral features confirm the successful incorporation of nHA into the CTS matrix. Notably, upon exosome loading, no significant spectral shifts or emergence of new absorption peaks were observed in either scaffold formulation, indicating that exosome incorporation does not disrupt the fundamental chemical structure of the composite.

#### 3.3.2. Degradation Behavior

The in vitro enzymatic degradation profiles of both scaffold types upon exposure to lysozyme (10 U/mL) over 4 weeks are shown in Figure 2E. Although both groups exhibited progressively increasing weight loss over time, the degradation rate of pure CTS scaffolds was markedly faster than that of CTS/10% nHA composites. This attenuation in degradation kinetics can be attributed to the water-insoluble nHA particles, which effectively reduce the contact area between the CTS pore walls and the enzymatic solution, thereby limiting the accessibility of lysozyme to glycosidic bonds within the chitosan matrix [32]. It is well established that a mismatch between scaffold degradation and new bone formation rates constitutes a major challenge in bone tissue engineering, as premature degradation leads to mechanical failure before sufficient bone ingrowth occurs, whereas excessively slow degradation impedes tissue remodeling [44]. Crucially, the moderated degradation rate of the CTS/10% nHA composite closely matches the natural pace of new bone tissue ingrowth, thereby preventing premature mechanical failure prior to structural bone regeneration [45].

#### 3.3.3. Swelling Behavior

The swelling performance of CTS/10% nHA scaffolds was evaluated in three different media: deionized water, PBS, and SBF (Figure 2F). Scaffolds reached swelling equilibrium in water and PBS within 12 h, achieving swelling ratios of 2855.9 ± 5.4% and 2886.6 ± 56.1%, respectively, whereas swelling equilibrium was not attained in SBF within the same timeframe. This delayed swelling response in SBF is likely attributable to ionic interactions between the inorganic ions present in SBF and the nHA particles, whose smaller particle size and correspondingly larger specific surface area result in a slower rate of solvent penetration [46]. The high swelling capacity observed in aqueous media indicates excellent hydrophilicity and fluid absorption, both of which are favorable properties for nutrient exchange and cell infiltration within the scaffold in vivo.

#### 3.3.4. Mechanical Properties

As presented in Figure S9, incorporating 10% nHA significantly enhanced the mechanical properties of the CTS scaffold. The compressive strength increased from 1.18 ± 0.02 to 1.34 ± 0.05 MPa, and the elastic modulus showed an approximately 2.5-fold improvement (from 11.03 ± 1.13 to 27.25 ± 2.93 MPa). This reinforcement is attributed to effective interfacial interactions between the rigid nHA nanoparticles and the chitosan chains, facilitating efficient stress transfer [47]. However, the breaking strain decreased substantially from 2.33 ± 0.16% to 1.10 ± 0.11%, indicating that nHA incorporation significantly embrittles the scaffold—a trade-off that must be carefully considered for clinical applications involving mechanical loading.

#### 3.3.5. Exosome Release Kinetics

The cumulative exosome release profile from the CTS/10% nHA scaffold demonstrated a characteristic biphasic pattern consisting of an initial burst release followed by sustained release over subsequent days (Figure 2G), with cumulative protein release reaching 89.73% by day 14. This release behavior carries significant implications for clinical translation, as it is well documented that naked exosomes injected directly into the body are rapidly cleared by the mononuclear phagocyte system (MPS) and the reticuloendothelial system—typically within hours—which severely limits their localized therapeutic efficacy [48]. Although previous studies have repeatedly highlighted the regenerative potential of mesenchymal stem cell-derived extracellular vesicles (MSC-EVs), their rapid clearance by bodily fluids and macrophages has remained a critical translational barrier [49]. In the present study, the CTS/10% nHA scaffold effectively functions as a protective physical niche that provides localized, sustained delivery of exosomal biological cues over a two-week period, thereby maximizing the therapeutic window for bone repair [50]. The initial burst release phase is advantageous for rapidly establishing a biologically active microenvironment at the defect site, while the subsequent sustained release ensures prolonged osteoinductive signaling to recruited progenitor cells during the critical early phase of bone healing [51].

#### 3.3.6. Cytocompatibility Assessment

Biocompatibility is a prerequisite for the clinical application of any scaffold material. The cytocompatibility of CTS/10% nHA scaffolds was evaluated using a Cell Counting Kit-8 (CCK-8) assay over a 2-day culture period (Figure 2H), and the results demonstrated that BMSCs cultured in the scaffold leaching solution exhibited continuous proliferation comparable to that of the control group (DMEM), confirming excellent cytocompatibility. This finding is consistent with the well-documented biocompatible nature of both chitosan and hydroxyapatite, which are recognized for their non-toxic degradation products and favorable cell–material interactions [52]. Taken together, these physicochemical and biological characterization results demonstrate that the CTS/10% nHA composite scaffold possesses an appropriate degradation rate, a well-interconnected porous structure, sustained exosome release capability, and excellent cytocompatibility, collectively supporting its potential as a multifunctional platform for exosome-mediated bone tissue regeneration.

### 3.4. Effects of HUCB-MSCs-Exos on Migration Ability and Osteogenic Differentiation of BMSCs

A fundamental prerequisite for any biomaterial system in bone tissue engineering is its capacity to promote the proliferation, migration, and osteogenic commitment of mesenchymal stem cells [53]. Consequently, we systematically evaluated the in vitro biological effects of HUCB-MSCs-exos on BMSCs.

**Figure 3:**
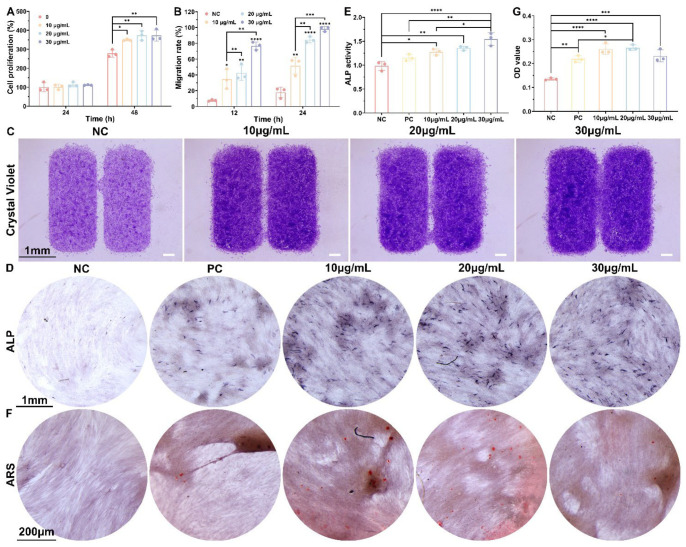
HUCB-MSCs-exos significantly promoted proliferation, migration, and osteogenesis differentiation. (**A**) Quantitative analysis of BMSCs cell proliferation. (**B**) Quantification of scratch wound closure in the scratch test. Scale bar, 1 mm. (**C**) Crystal violet staining after 24 h of migration. (**D**) Representative images of ALP staining of MC 3T3-E1 treated with HUCB-MSCs-exos. Scale bar, 1 mm. (**E**) Quantification of ALP staining. (**F**) Representative images of ARS staining of MC 3T3-E1 treated with HUCB-MSCs-exos. Scale bar, 200 µm. (**G**) Quantification of ARS staining. (* *p* < 0.05, ** *p* < 0.01, *** *p* < 0.001, **** *p* < 0.0001).

The proliferative effects of HUCB-MSCs-exos on BMSCs were assessed using a CCK-8 assay. After 24 h, no significant inter-group differences were observed, suggesting that a latency period is required for exosome internalization and the subsequent activation of intracellular signaling pathways. By 48 h, however, the results revealed that HUCB-MSCs-exos significantly promoted BMSC proliferation in a concentration-dependent manner, with the 20 and 30 μg/mL treatment groups exhibiting the highest proliferative activity (Figure 3A). This dose-dependent response aligns with previous findings that exosome concentrations ranging from 20 to 50 μg/mL create an optimal microenvironment for sustaining stromal cell metabolic activity without inducing cellular senescence or overstimulation [54]. The pro-proliferative effects of exosomes are often attributed to the horizontal transfer of functional microRNAs and growth factors, which can activate signaling cascades such as the mitogen-activated protein kinase/extracellular signal-regulated kinase (MAPK/ERK) pathway, thereby accelerating the G1/S phase transition of the cell cycle [55].

Cell migration, a critical initial step in the bone healing cascade involving the recruitment of endogenous progenitor cells [49], was evaluated via a scratch wound-healing assay. After 24 h, crystal violet staining demonstrated that exosome-treated groups exhibited markedly enhanced cellular repopulation of the scratch area compared to the negative control (DMEM only), indicating superior migratory capability (Figure 3B,C and Figure S10). Notably, the deeper color intensity observed in the non-scratch regions of these treated groups further corroborated the enhanced proliferative activity reported by the CCK-8 assay (Figure 3A).

To evaluate osteogenic potential, a crucial determinant of bone regeneration [56], we employed ALP staining and ARS staining as respective markers for early and late-stage osteogenesis [57]. Qualitative analysis of ALP staining revealed that while the negative and positive control groups showed relatively light staining, the addition of HUCB-MSCs-exos at various concentrations resulted in progressively deeper staining (Figure 3D). Quantitative analysis corroborated this observation, identifying the 20 μg/mL concentration as the most effective for promoting early osteogenic activity (Figure 3E). Subsequently, quantitative ARS analysis after 21 days of culture demonstrated significantly enhanced extracellular matrix mineralization in the exosome-treated groups, with the 30 μg/mL concentration yielding the most substantial mineral deposition (Figure 3F,G). This slight discrepancy between the optimal concentrations for ALP activity (early stage) and mineralization (late stage) may reflect the differential dose requirements for distinct phases of osteogenic maturation [58]. This robust enhancement of osteogenesis is likely attributable to the rich cargo of osteoinductive molecules, such as microRNAs (e.g., miR-196a, miR-21) and proteins (e.g., BMP-2, TGF-β), encapsulated within the exosomes. These molecules are known to activate key osteogenic signaling pathways, including Wnt/β-catenin and PI3K/Akt, and upregulate master transcription factors like RUNX2 and Osterix (SP7) (unpublished data), thereby driving the osteoblastic lineage commitment of recipient BMSCs [59].

### 3.5. Promoted Bone Regeneration by CTS/10% nHA-Exo In Vivo

Having demonstrated the strong pro-osteogenic potential of HUCB-MSCs-derived exosomes in vitro, we next evaluated their therapeutic efficacy in vivo using a composite scaffold–based delivery system. A rabbit condylar defect model (5 mm in diameter) was established, and animals were randomly assigned to five groups: NC (untreated), CTS, CTS/10% nHA, CTS/10% nHA-exo, and PC (bone cement). In this model, a 5-mm defect is considered critical-sized, meaning that spontaneous bone healing does not occur within the experimental observation period, thereby providing a stringent and reliable platform for assessing the regenerative performance of biomaterial-based interventions [60].

**Figure 4:**
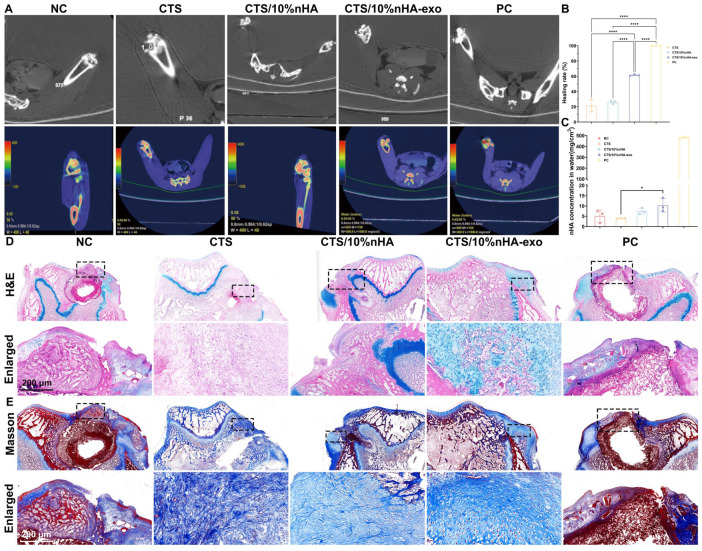
Bone regeneration capability of CTS/10% nHA-exo in condyle rabbit bone-defect models. (**A**) CT reconstructions of the defects after 1-month post-surgery in different groups. (**B**) The healing rate was determined by CT and normalized against the NC group. (**C**) The concentration of nHA in water in different groups. (* *p* < 0.05, **** *p* < 0.0001). (**D**) H&E staining and (**E**) Masson’s trichrome staining for bone defect (detailed images of the defect areas within the black boxes). Scale bar, 200 μm.

Two months after surgery, CT imaging revealed minimal new bone formation in both the NC and CTS groups. The CTS/10% nHA group exhibited limited bone regeneration primarily at the margins of the defect, indicating that although the osteoconductive scaffold provides a structural substrate for cell attachment and migration, its intrinsic regenerative stimulus remains insufficient. In contrast, the CTS/10% nHA-exo group displayed markedly enhanced osteogenic regeneration compared with all other experimental groups (Figure 4A). In the PC (bone cement) group, no apparent bone defect was observed in CT images due to the inherent radiopacity and space-filling characteristics of bone cement; however, this radiographic appearance reflects mechanical filling of the defect rather than genuine biological bone regeneration.

Quantitative morphometric analysis further confirmed these observations. At two months post-implantation, the CTS/10% nHA-exo group achieved a significantly higher healing rate (57.44 ± 8.42%) than both the CTS group (21.42 ± 7.93%) and the CTS/10% nHA group (29.33 ± 6.94%) (Figure 4B). This nearly two-fold increase in new bone formation compared with conventional CTS/nHA composites—which typically produce only 30–40% defect closure at similar time points [61]—provides strong evidence for the enhanced in vivo regenerative efficacy mediated by exosome-derived paracrine signaling. Consistently, the CTS/10% nHA-exo group also exhibited the highest nHA content at the defect site among all groups (Figure 4C), suggesting active mineral deposition and effective incorporation of nHA particles into the newly formed bone matrix.

Bone regeneration across all experimental groups was quantitatively assessed at two months post-surgery, revealing that the CTS/10% nHA-exo group exhibited markedly superior bone regeneration compared to all other groups (Figure 4A, Table S1). Its bone volume fraction (BV/TV) reached 93.57 ± 2.93%, significantly exceeding the NC (66.12 ± 4.53%), CTS (74.13 ± 1.94%), PC (71.67 ± 0.11%), and CTS/10% nHA (78.90 ± 2.03%) groups (all *p* < 0.001), approaching near-complete defect closure and confirming the critical-sized nature of the defect model. Trabecular microarchitectural analysis further corroborated these findings. The CTS/10% nHA-exo group exhibited the lowest trabecular separation (Tb.Sp, 4.50 ± 2.12 μm), both significantly different from other groups (*p* < 0.01), indicative of densely packed, well-integrated new bone. Notably, despite achieving the highest BV/TV, this group showed a relatively lower trabecular thickness (Tb.Th, 46.20 ± 7.41 μm) compared to the CTS/10% nHA group (Tb.Th, 82.02 ± 9.40 μm). This pattern of numerous thin trabeculae at high density rather than fewer thick ones—is characteristic of active early-stage osteogenesis and reflects a dynamic remodeling state [62].

### 3.6. Histological and Morphological Evaluation of In Vivo Bone Regeneration

To obtain detailed insights into the quality and maturity of the regenerated tissue beyond radiographic assessment, macroscopic and histological examinations were performed at 2-month post-surgery (Figure S10 and Figure 4D,E).

In the NC group, the defect area was predominantly filled with fibrous hyperplastic tissue, indicating a fibrotic healing response rather than true osteogenesis (Figure S11). The bone cement (PC) group displayed distinct internal calcified foci with superficial newly formed cartilage, suggesting limited endochondral ossification confined strictly to the cement–tissue interface. For the CTS and CTS/10% nHA groups, although the scaffold materials had not completely degraded, their surfaces were covered with a thin layer of newly formed cartilage, marking the onset of the endochondral ossification cascade. Strikingly, in the CTS/10% nHA-exo group, the implanted scaffold was completely resorbed and replaced by well-organized, newly formed trabecular bone replete with blood sinuses. This synchronization between scaffold degradation and functional bone tissue ingrowth represents the ideal paradigm for degradable bone tissue engineering [63].

H&E and Masson’s trichrome staining further corroborated these macroscopic findings (Figure 4D,E). In the CTS/10% nHA-exo group, H&E staining revealed intact, mature bone trabeculae filling the defect core. The surface was covered by hyperplastic cartilage exhibiting robust Alcian blue positivity, indicating active proteoglycan deposition characteristic of vigorous endochondral ossification. In marked contrast, the NC, CTS, and PC groups exhibited extensive muscle fiber infiltration (stained red in Masson’s trichrome) within the cartilaginous areas, with no discernible trabecular bone in the central defect—a hallmark of arrested repair. The CTS/10% nHA group displayed abundant Alcian blue-positive staining but retained residual muscle fibers, indicating that trabecular bone reconstruction was still incomplete. This intermediate histological phenotype underscores that while the CTS/10% nHA composite provides excellent osteoconductivity, it is insufficient to achieve complete defect healing within this timeframe. The osteoinductive contribution of exosomes is thus indispensable for driving the ossification process to completion.

A pivotal observation in the CTS/10% nHA-exo group was the abundance of functional blood sinuses integrated within the newly formed bone. It is well established that the spatial and temporal coupling of angiogenesis and osteogenesis (type H vessels) is a vital prerequisite for the long-term survival and remodeling of regenerated bone [64]. Functional vascularization is essential not only for oxygen and nutrient delivery but also for recruiting osteoprogenitor cells. The pronounced vascularization specifically observed in the exosome-functionalized group strongly indicates that HUCB-MSCs-exos potently stimulate angiogenesis. This is likely mediated through the sustained paracrine delivery of pro-angiogenic cargos, such as VEGF and miR-210, which synergistically promote endothelial cell tube formation and vessel maturation at the defect site [65].

### 3.7. Immunohistochemical Validation of Osteogenic Maturation

**Figure 5:**
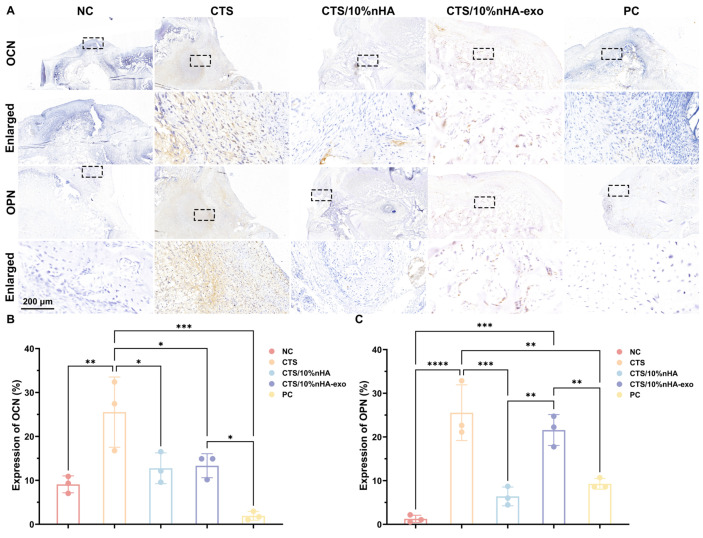
Immunohistochemical analyses for OCN and OPN in condyle rabbit bone-defect models. (**A**) Representative images of immunohistochemistry staining (Detailed images of the positive expression within the black boxes). Scale bar, 200 μm. Quantitative analysis of the expression of (**B**) OCN and (**C**) OPN. (* *p* < 0.05, ** *p* < 0.01, *** *p* < 0.001, **** *p* < 0.0001).

To molecularly dissect the quality of the newly formed bone matrix, immunohistochemistry was employed to evaluate the expression of two definitive late-stage osteogenic markers: OCN and OPN (Figure 5A). OCN is a non-collagenous protein specifically secreted by mature osteoblasts, directly correlating with bone formation activity and matrix mineralization [66]. OPN is a multifunctional phosphoprotein that mediates cell–matrix interactions at the mineralization front and regulates dynamic bone remodeling [67].

Quantitative IHC analysis revealed a biologically informative gradient of osteogenic activity across groups (Figure 5B). The PC group showed near-absent staining for both markers (OCN ~2%; OPN ~9%), consistent with the biological inertness of calcium phosphate cement, which provides structural occlusion but fails to sustain osteoprogenitor differentiation [68]. The NC group showed low OCN (~9%) but negligible OPN (~1%), reflecting baseline fibrous healing rather than true osteogenic regeneration. Unexpectedly, the CTS group exhibited the highest expression of both OCN (~25%) and OPN (~26%), with widespread brownish IHC staining, indicating that chitosan alone creates a highly permissive microenvironment for osteoblast recruitment—likely via its pro-inflammatory resolution properties, favorable porosity, and preservation of endogenous growth factor gradients [69]. However, these elevated markers must be interpreted alongside the notably low collagen content in this group (~54%) (Figure S12), indicating an active yet structurally immature remodeling phase in which osteoblasts have not yet deposited a mechanically competent organic matrix.

The CTS/10%nHA group showed paradoxically reduced OCN (~13%) and OPN (~6%) despite nHA’s established osteoconductive properties. This likely reflects how nHA-induced changes in scaffold stiffness and surface chemistry shift local biophysical cues toward a more quiescent osteogenic state at the 2-month timepoint, delaying peak marker expression [70]. Notably, collagen content recovered to ~70%—approaching the NC baseline—suggesting that suppressed marker activity coincided with substantially more mature matrix organization relative to CTS alone (Figure S12). The CTS/10%nHA-exo group presented a distinctive profile: OCN remained moderate (~13%), while OPN was markedly elevated (~21%), significantly exceeding the CTS/10%nHA group (*p* < 0.01). This selective OPN upregulation suggests that HUCB-MSC-derived exosomes specifically potentiate the matrix maturation and cell–matrix interaction phases of osteogenesis rather than uniformly amplifying all osteogenic markers. Exosomal cargo—including osteo-regulatory microRNAs (e.g., miR-2861, miR-335-5p) and bioactive proteins—likely acts through targeted activation of the RUNX2–OPN axis, enhancing the adhesive and organizational capacity of the nascent bone tissue [71]. The concurrent high collagen content (~70%) further confirms that the exosome-functionalized scaffold supports a structurally mature organic matrix, a prerequisite for long-term mechanical competence.

Collectively, these findings reveal a nuanced hierarchy of osteogenic quality rather than a simple dose–response relationship. The CTS/10%nHA-exo group—characterized by selective OPN elevation alongside mature collagen deposition—most closely approximates a physiologically coordinated remodeling state, wherein the matrix is simultaneously mineralizing and structurally consolidating. This contrasts with the CTS group, which, despite peak OCN and OPN levels, lacks the collagen matrix maturity required for functional bone quality. These results affirm that HUCB-MSC-derived exosomes fine-tune the spatiotemporal coordination of bone matrix maturation, transforming the CTS/10%nHA scaffold from a passive osteoconductive carrier into an active, matrix-organizing osteoinductive platform.

### 3.8. Limitations and Future Directions

Despite the highly promising osteo-angiogenic outcomes demonstrated in this study, several important limitations warrant explicit acknowledgment. First, the rabbit condylar defect model employed herein represents a non-load-bearing experimental environment; although this model is well established for evaluating baseline biocompatibility and early-stage osteogenesis, it does not adequately replicate the complex biomechanical stress conditions characteristic of human long bone defects—such as those encountered in femoral or condyle fractures. Critically, the absence of physiological mechanical loading may subtly but meaningfully alter scaffold degradation kinetics and bone remodeling dynamics relative to real clinical scenarios, in which Wolff’s law-driven mechanotransduction plays a pivotal and indispensable role in directing mature bone architecture [72]. Second, although robust macroscopic and histological healing driven by exosome functionalization was clearly observed, the precise stoichiometric contributions of specific exosomal microRNAs versus cargo proteins to the osteogenic and angiogenic outcomes remain incompletely characterized, representing a significant mechanistic gap that future investigations must address. Third, the single observational window spanning only one to two months substantially limits the capacity to assess long-term bone remodeling trajectories, scaffold–tissue integration fidelity, and the sustained durability of the regenerative outcomes achieved.

In light of these limitations, future research should prioritize several interconnected and scientifically consequential directions. To begin with, evaluating this exosome-functionalized CTS/nHA scaffold in larger, load-bearing animal models—such as sheep metatarsal or minipig mandibular defect models—is essential for confirming its mechanical durability, structural integrity under physiological stress, and ultimate translational viability for orthopedic and maxillofacial surgical applications [73]. Furthermore, deeper transcriptomic and proteomic profiling of the exosomal cargo, employing advanced methodologies such as single-cell RNA sequencing and mass spectrometry-based proteomics, will be necessary to isolate and precisely define the functional exosomal components—including specific let-7 or miR-21 microRNA clusters—that orchestrate the paracrine crosstalk between endothelial cells and osteoblasts during coupled angiogenesis and osteogenesis. Finally, future studies should incorporate substantially extended follow-up periods of six to twelve months to rigorously evaluate the quality, maturation, and functional competence of the regenerated bone tissue, encompassing its biomechanical properties, cortical–cancellous architectural organization, and degree of functional integration with the surrounding native bone [74].

## 4. Conclusions

In conclusion, this study successfully addresses a critical challenge in regenerative medicine by developing an exosome-functionalized CTS/10% nHA nanoscaffold that effectively bridges the gap between passive mechanical support and active biological induction. Through the sustained release of HUCB-MSC-derived exosomes over a 14-day period, the composite scaffold mitigates rapid exosome clearance while ensuring prolonged bioactive signaling at the defect site. Quantitative analyses revealed a remarkable in vivo bone healing rate of 57.44% at one month post-implantation, significantly outperforming that of conventional acellular scaffolds. Complementary histological and immunohistochemical evaluations further corroborated the formation of well-organized trabecular bone accompanied by extensive vascularization in the exosome-functionalized group. Collectively, the synergistic interplay between the osteoconductivity of the CTS/nHA matrix and the potent osteo-angiogenic paracrine signaling mediated by the loaded exosomes substantiates the therapeutic superiority of this hybrid approach, thereby establishing it as a highly viable, cell-free strategy for the targeted repair of critical-sized bone defects.

## Figures and Tables

**Table 1:** Immunohistochemistry reagents.

Primary Antibody	Clonality	Optimal Dilution	Blocking Serum	Secondary Antibody
OPN	Rabbit monoclonal	1:100	Goat	Goat anti-rabbit
OCN	Mouse monoclonal	1:100	Goat	Goat anti-mouse

## Data Availability

The original contributions presented in this study are included in the article/Supplementary Material. Further inquiries can be directed to the corresponding authors.

## Supplementary Materials

The following supporting information can be downloaded at: https://www.mdpi.com/article/10.3390/polym18131562/s1.
