# Supplementary material for: Enhanced Bone-Defect Regeneration Through nHA/Chitosan Nanocomposite-Facilitated Delivery of HUCB-MSCs-Derived Exosomes
Source: Polymers (Basel). 2026 Jun 23;18(13):1562. doi: 10.3390/polym18131562 (PMC13364372; doi:10.3390/polym18131562)
Supplement: Supplementary file 1 [file polymers-18-01562-s001.zip › polymers-4337472-supplementary.pdf]

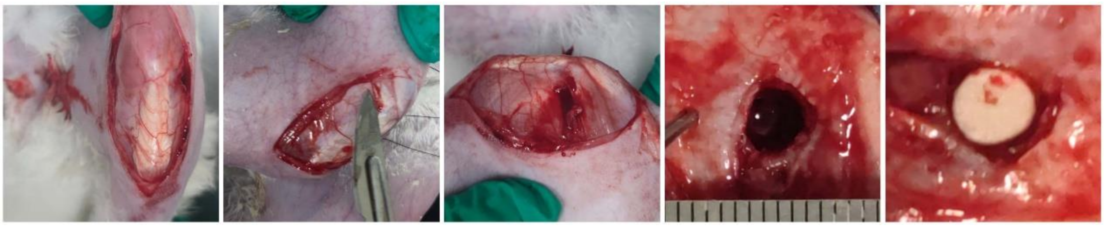

Figure S1. Intraoperative image of the composite scaffold implanted into a critical defect of the rabbits.

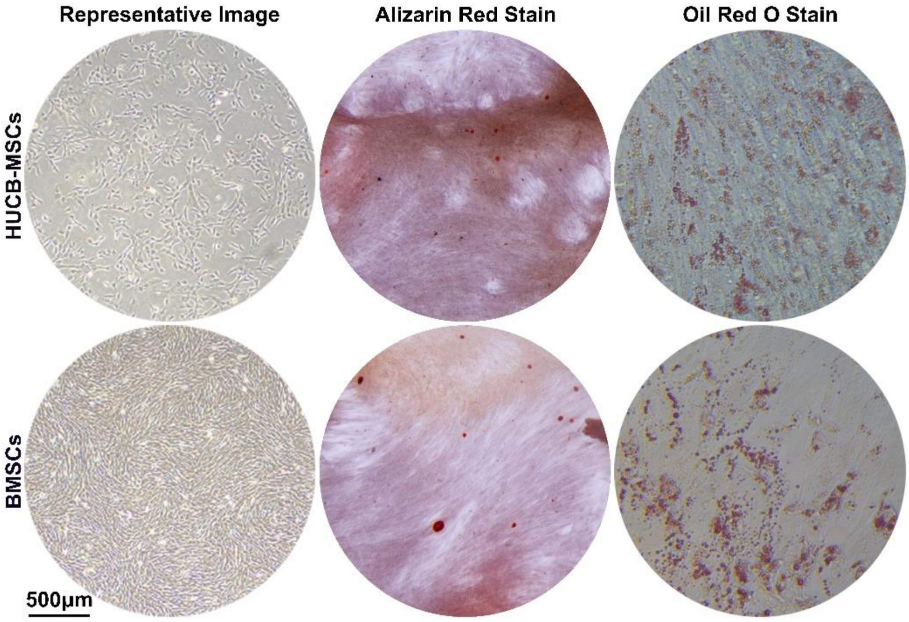


Figure S2. Representative images of HUCB-MSCs and BMSCs morphological characteristics. Scale bar, 500 μm.

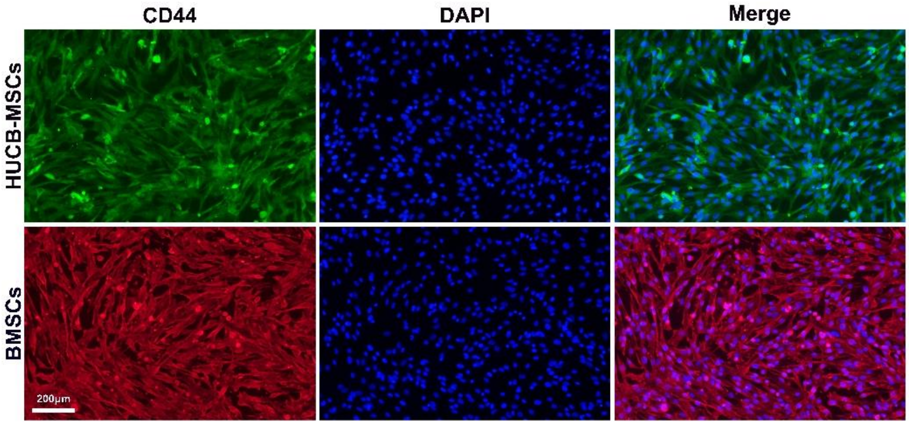


Figure S3. Immunofluorescence identification of HUCB-MSCs and BMSCs. Scale bar, 200 μm.

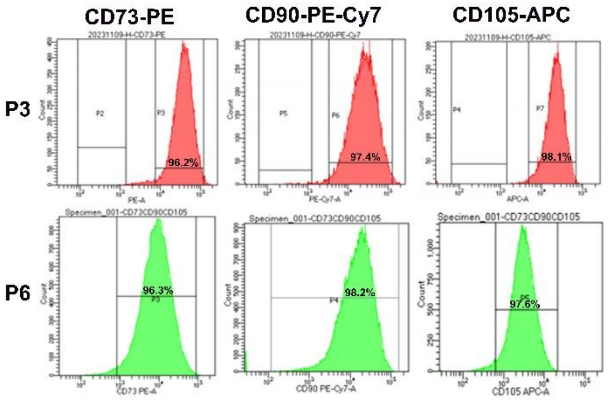


Figure S4. Flow cytometry showing positive P3 and P6 of HUCB-MSC surface markers.

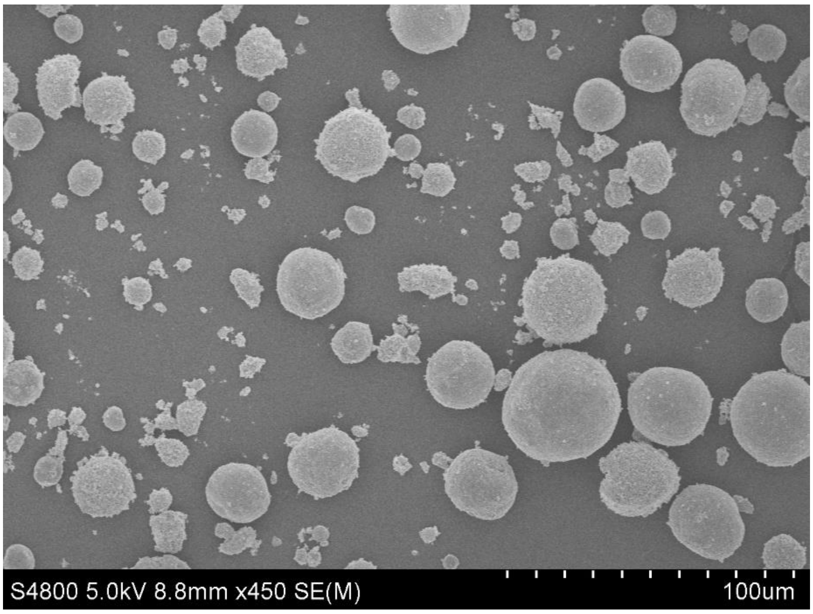


Figure S5. SEM image of nHA. Scale bar, 100 μm.

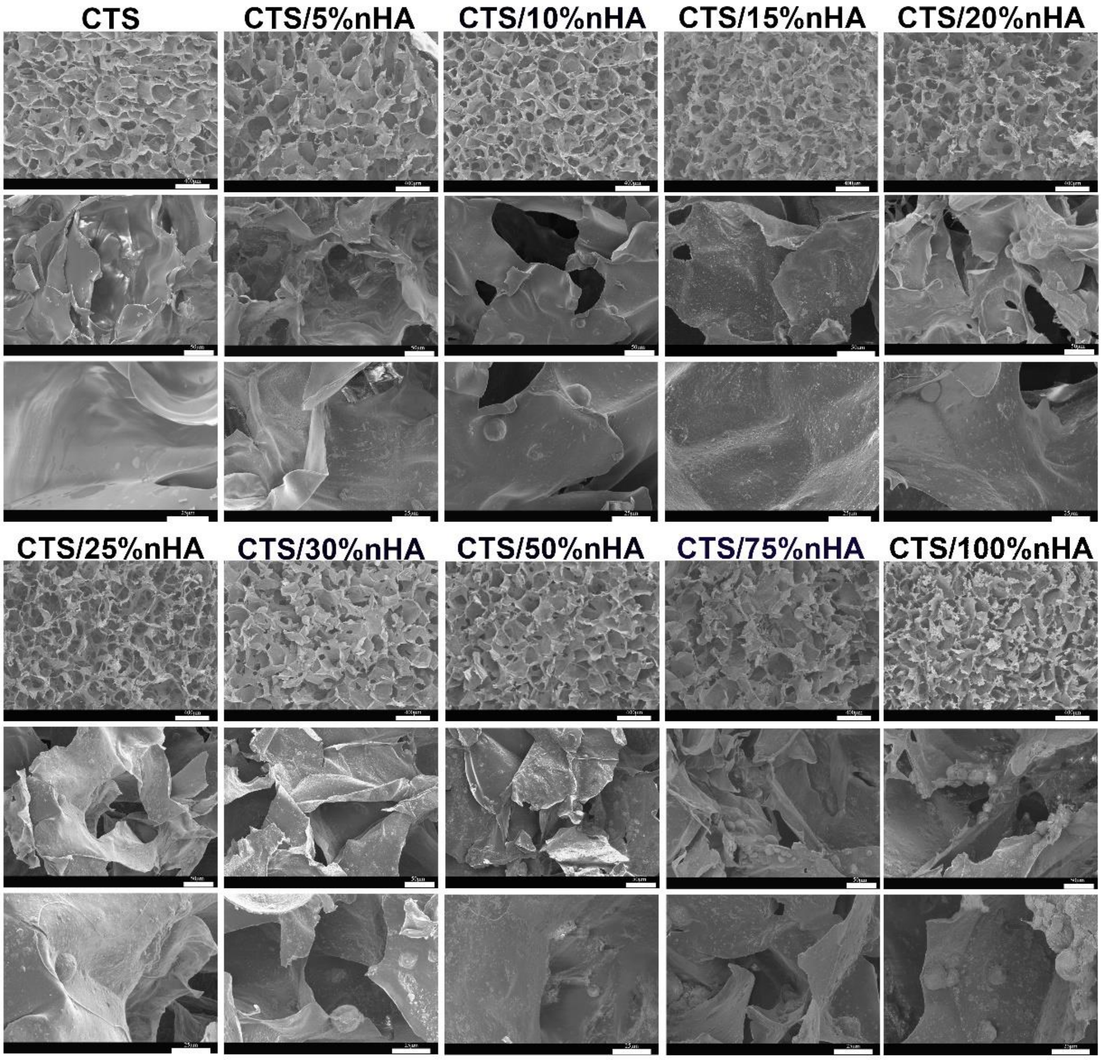


Figure S6. SEM image of various combinations of CTS and nHA at different ratios. Scale bar, 400 μm, 50 μm and 25 μm, respectively.

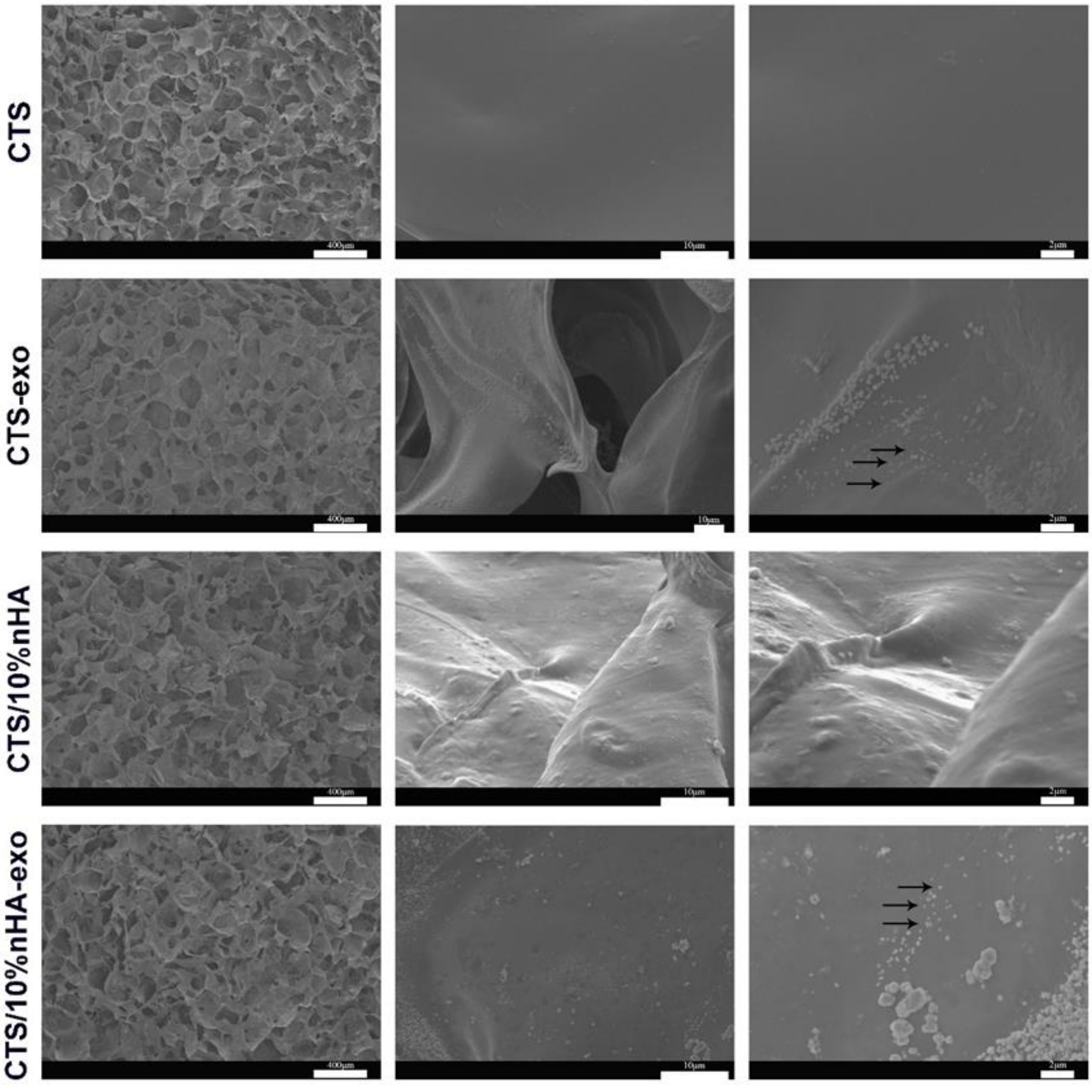


Figure S7. SEM images of scaffolds with or without exosomes. The black arrow represents exosomes loaded on the scaffold. Scale bar, 400 μm, 10 μm and 2 μm, respectively.

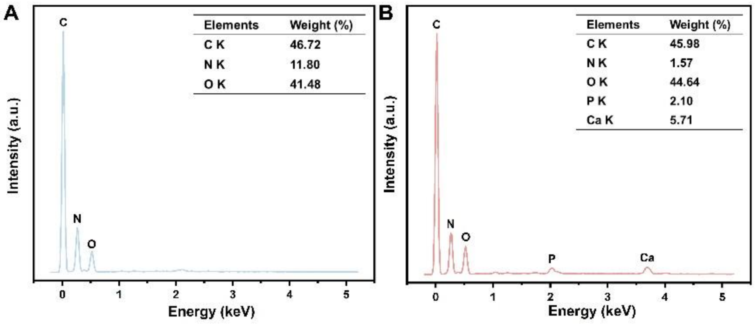


Figure S8. EDS assay of (A) CTS and (B) CTS/10% nHA.

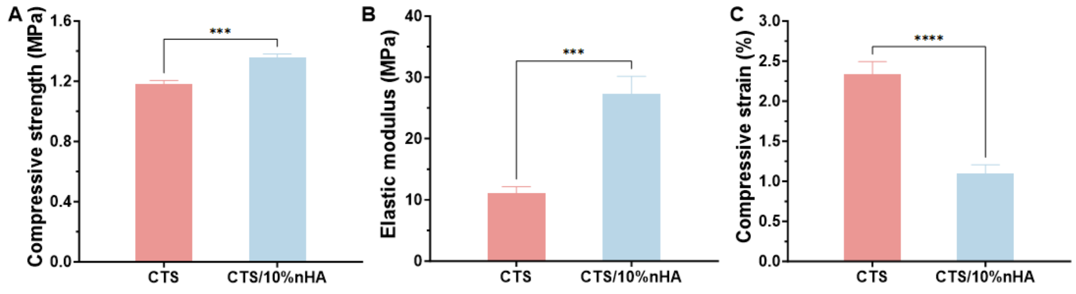


Figure S9. Mechanical properties of CTS and CTS/10% nHA scaffolds. (A) Compressive strength, (B) elastic modulus, and (C) compressive strain of CTS and CTS/10% nHA scaffolds. ($^{***}$ $P$ < 0.001, $^{****}$ $P$ < 0.0001).

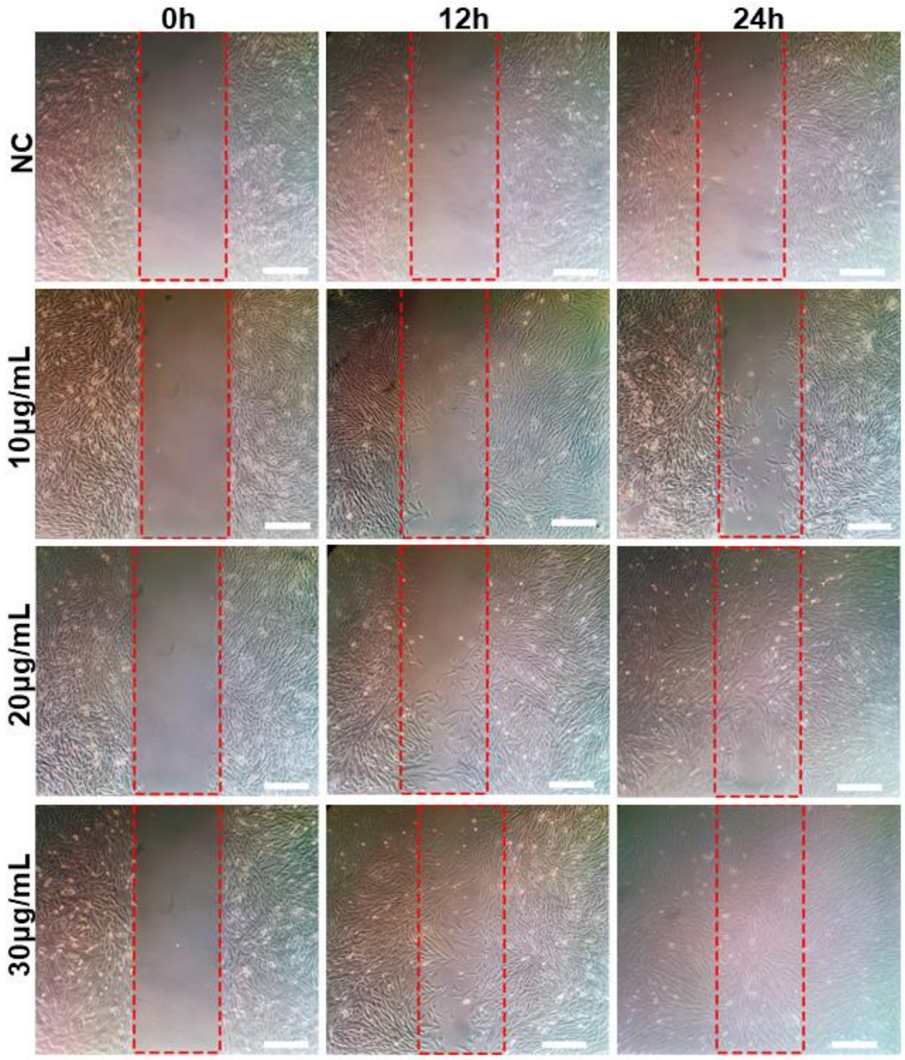


Figure S10. Representative images of the scratch test co-cultured BMSCs with HUCB-MSCs-exos. The red dotted rectangles indicate the initial boundary of the wounds. Scale bar, 250 μm.

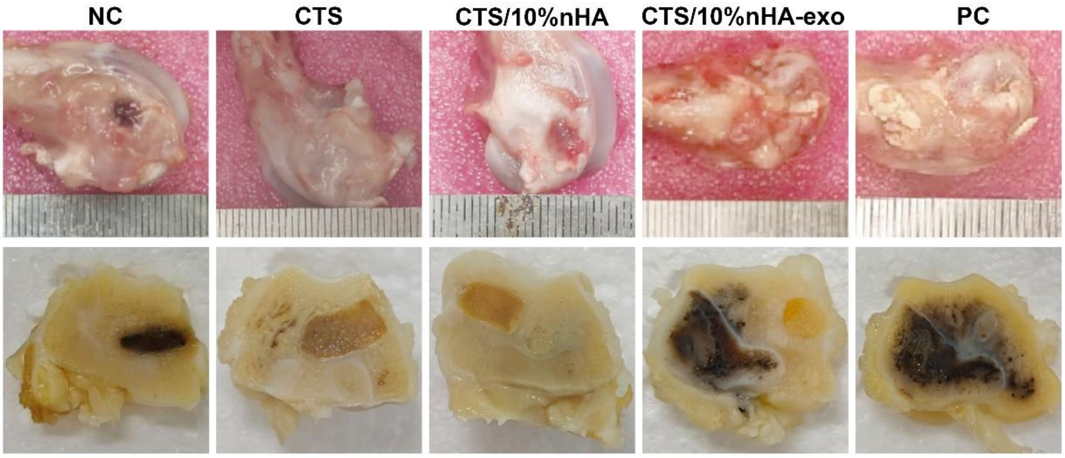


Figure S11. Representative images of different groups after euthanasia and decalcification of bone tissue.

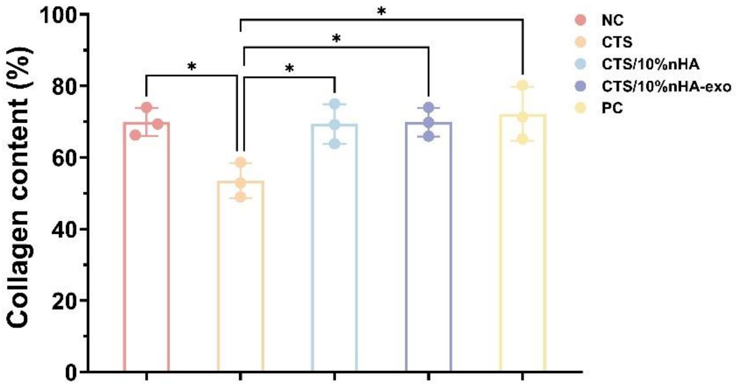


Figure S12. Quantitative analysis of collagen content (* $P < 0.05$).

Table S1. Quantitative analysis of bone regeneration parameters ($^{*}$ $P < 0.05$, $^{**}$ $P < 0.01$, $^{***}$ $P < 0.001$).

| **Parameters** | **NC** | **CTS** | **CTS/10%nHA** | **CTS/10%nHA/exo** | **PC** |
|---|---|---|---|---|---|
| BV/TV (%) | 66.12 ± 4.53 | 74.13 ± 1.94$^{*}$ | 78.90 ± 2.03** | **93.57** ± **2.93**$^{***}$ | 71.67 ± 0.11 |
| Tb.Th (μm) | 60.05 ± 15.46 | 67.32 ± 12.46 | 82.02 ± 9.40 | 46.20 ± 7.41 | 67.60 ± 2.19 |
| Tb.Sp (μm) | 26.43 ± 5.14 | 24.90 ± 1.91 | 26.08 ± 1.97 | **4.50** ± **2.12**$^{***}$ | 28.83 ± 1.13 |
